# Droplet-Based Microfluidics: Applications in Pharmaceuticals

**DOI:** 10.3390/ph16070937

**Published:** 2023-06-28

**Authors:** Thi Ngoc Diep Trinh, Hoang Dang Khoa Do, Nguyen Nhat Nam, Thach Thi Dan, Kieu The Loan Trinh, Nae Yoon Lee

**Affiliations:** 1 Department of Materials Science, School of Applied Chemistry, Tra Vinh University, Tra Vinh City 87000, Vietnam; 2 NTT Hi-Tech Institute, Nguyen Tat Thanh University, Ward 13, District 04, Ho Chi Minh City 70000, Vietnam; 3 Biotechnology Center, School of Agriculture and Aquaculture, Tra Vinh University, Tra Vinh City 87000, Vietnam; 4 BioNano Applications Research Center, Gachon University, 1342 Seongnam-daero, Sujeong-gu, Seongnam-si 13120, Republic of Korea; 5 Department of BioNano Technology, Gachon University, 1342 Seongnam-daero, Sujeong-gu, Seongnam-si 13120, Republic of Korea

**Keywords:** droplet, microfluidics, drug discovery, drug screening, drug delivery, spheroids, cell culture

## Abstract

Droplet-based microfluidics offer great opportunities for applications in various fields, such as diagnostics, food sciences, and drug discovery. A droplet provides an isolated environment for performing a single reaction within a microscale-volume sample, allowing for a fast reaction with a high sensitivity, high throughput, and low risk of cross-contamination. Owing to several remarkable features, droplet-based microfluidic techniques have been intensively studied. In this review, we discuss the impact of droplet microfluidics, particularly focusing on drug screening and development. In addition, we surveyed various methods of device fabrication and droplet generation/manipulation. We further highlight some promising studies covering drug synthesis and delivery that were updated within the last 5 years. This review provides researchers with a quick guide that includes the most up-to-date and relevant information on the latest scientific findings on the development of droplet-based microfluidics in the pharmaceutical field.

## 1. Introduction

Drug development is one of the most important science fields that contribute to the improvement of human health. Drug development maximizes the bioactivity of drug compounds and reduces side effects in the body, thereby improving treatment, fighting infections, and prolonging human life [1]. Moreover, developing new drugs helps reduce the time and expense of healthcare [2,3,4]. Typically, drug development includes drug synthesis, screening, delivery, and evaluation [5]. First, the target compound is selected, synthesized, and subjected to various screening steps to increase drug applicability. Second, a drug delivery strategy is selected to load the drug and deliver it to the target sites in the body to achieve therapeutic effects. Third, drug evaluation assesses the activity, toxicity, and pharmacokinetics of drugs [6]. Drug development requires efficient and rapid methods for screening, synthesizing, delivering, and evaluating drug candidates. However, this is a costly, time-consuming, sophisticated, and massive process. For example, drug screening is usually performed using platforms such as 96-well plates, which is a time-consuming task with a low throughput [4]. Drug development requires cell culture and animal testing. Typically, a monolayer cell culture is employed, but this does not reflect the results of later testing steps, such as clinical testing, because the cell layer in 2D in vitro models is very different from that in 3D in vivo models [4]. Furthermore, animal experiments are expensive and time-consuming, and are strictly regulated depending on the region [7]. In addition, animal models are not always suitable for drug testing because of the differences between animal and human bodies [8,9,10]. Although a large number of drug candidates are discovered every year, only a few obtain permission to enter later drug trials and obtain final approval for use [11,12]. Therefore, new approaches for improving drug development are crucial for the pharmaceutical industry.

As a subset of microfluidic technologies, droplet-based microfluidic devices offer potential solutions for drug development [13,14]. Microfluidics manipulate the fluid flow inside a microchannel system [15]. Droplet-based microfluidics generate and manipulate monodisperse drops ranging from femtoliters to nanoliters in volume within an immiscible phase [16,17]. This method benefits from its microscale nature and allows the integration of sample preparation, analysis, and detection with high-throughput and precise control. This method has many advantages over bulk reactions, such as a shorter reaction time, sample and reagent savings, high multiplexing, and a high sensitivity [18,19].

The use of droplet-based microfluidic devices for drug development offers several advantages over conventional methods [20]. Below, we list the advantages of droplet-based microfluidics in drug development. First, the advantage brought by the high surface-area-to-volume ratio of microfluidics can shorten the reaction time for drug development. For example, Schoepp et al. developed a droplet-based microfluidic system to evaluate the activity of antibiotics in 30 min [21]. The system, integrated with loop-mediated isothermal amplification, offers an ultrafast assay to evaluate the susceptibility of *Escherichia coli* (*E. coli*) to certain types of antibiotics. Second, droplet-based microfluidic devices offer multiplex assays for drug screening. For example, antimicrobial susceptibility testing benefits from the multiplex ability of droplet-based microfluidic devices. This system could be used to test multiple concentrations of antibiotics. A high-throughput droplet system was introduced to predict synergy between various antibiotics against *E. coli* [22]. Third, the droplet-based microfluidic system offers a controlled environment for drug testing of various types of cells, including bacteria and cancer cells. The system can isolate single cells and allow for the simple observation of cell responses under the effect of the applied drug. This provides valuable information for the development of new drugs [4].

We begin this review with a short description of drug development and discuss the need for new approaches to drug development. Then, we briefly introduce droplet-based microfluidic devices and their advantages for drug development applications. In the next section, we describe droplet-based microfluidics in terms of device fabrication, droplet generation, and droplet manipulation. We then explain how droplet-based microfluidics could be applied in drug development. Several studies have been published on droplet generation [23,24]. In this review, we introduce different methods and discuss their advantages and disadvantages to provide a clearer understanding of them. Finally, we arrive at the conclusion and discuss the existing problems of droplet-based microfluidics and possible future directions.

## 2. Droplet-Based Microfluidics

Microfluidics manipulate the fluid flow inside a microchannel system. Consequently, microfluidics deal with small fluid volumes. Droplet-based microfluidics, a branch of microfluidics, generate and manipulate monodispersed droplets in micron size [25,26,27].

### 2.1. Droplet-Based Microfluidic Device Fabrication

The structure of the device should be well designed to effectively control the fluid flow at microscale. The fabrication of droplet-based microfluidic devices employs various strategies to manufacture microfluidic chips [28]. To fabricate droplet-based microfluidic devices, a wide range of materials has been employed, including silicon, glass, and polymers [3]. Among these, polydimethylsiloxane (PDMS), an elastomer, is the most common material because it is flexible, optically transparent, and easily molded and bonded [29,30]. Polymer-based replica molding techniques are widely used because of their low cost, rapidity, and ease of processing. To fabricate the PDMS microfluidic chip, a master mold with the designed patterns is fabricated. PDMS is prepared by mixing the PDMS prepolymer and curing agent in the desired ratio. The mixture is then mixed well, degassed, poured over the master mix, and incubated at a high temperature. The cured PDMS is then removed from the master and replica. Micropatterns such as microchannels and chambers are replicated in PDMS. Finally, the micropatterned PDMS is bonded to another substrate to provide an intact microfluidic structure.

Meanwhile, 3D printing, also known as the layer-by-layer method for additive manufacturing systems, has recently been developed for microdevice fabrication using high-resolution, low-cost, and rapid prototyping techniques [31,32,33]. The 3D printing process is usually eco-friendly and promotes its industrialization, especially in the field of drug development [34,35,36]. Noroozi et al. fabricated a 3D-printed microfluidic droplet generation system for polycaprolactone (PCL) droplets loaded with dexamethasone for drug delivery applications using stereolithography (SLA) and fused deposition modeling (FDM) [37]. Using SLA printing, a 3D-printed mold with a resolution of 50 μm was successfully fabricated, and the assisted 3D-printed chip could generate PCL droplets with a higher encapsulation efficiency. In another study, Anyaduba et al. fabricated a picoliter droplet generation device for a biomolecule analysis using the 3D printing technique [38]. With inexpensive and simple instrumentation (a 3D printer and syringe pump), this device can generate monodisperse droplets as small as ~48 pL. In 2023, Shi et al. developed a single-chip silicone-integrated device for monodisperse picoliter droplets using a printing nozzle and T-junction as a droplet generator, which could achieve attomole-level (86 amol) detection sensitivity for the mass spectrometry imaging detection of GABA [39]. Although 3D printing shows high potential for microfluidic fabrication, this technique still requires optimization and improvement in terms of accuracy and resolution. The development of 3D printing has become a priority for microfluidic device fabrication on an industrial scale in the future.

### 2.2. Droplet Generation

**Figure 1:**
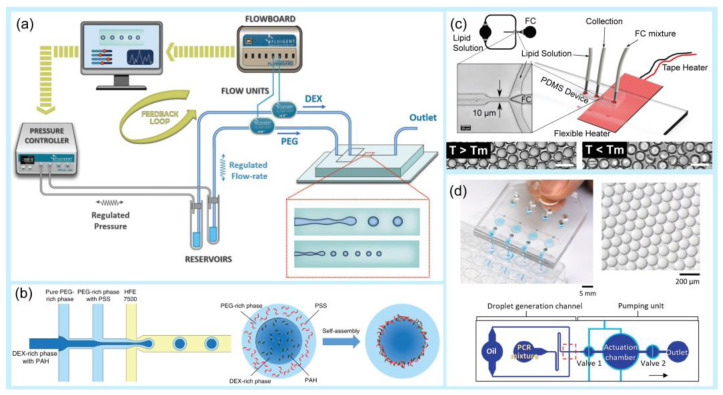
Examples of droplet generation techniques. (**a**) Co-flow. Experiment setup and schematic of a microfluidic chip for droplet generation. Variations in droplet size as a function of *P*_PEG_ at different *P*_DEX_. Reprinted with permission from [68]. Copyright (2018) ACS publications. (**b**) A multi-inlet flow-focusing channel generator. The morphology of double-emulsion drops formed at different core–shell flow-rate ratios (scale bar: 20 μm). Image of the double-emulsion drops generated at flow rates of 200 μL/h (DEX-rich), 600 μL/h (PEG-rich), and 3000 μL/h (oil). Scale bar: 100 μm. Reprinted with permission from [69]. Copyright (2020) Springer Nature. (**c**) Thermal control. Schematic of the microfluidic droplet chip and integrated heating system. Endoskeletal droplets generated using this technique at a higher temperature (*T* > *T*_m_) and a lower temperature (*T* < *T*_m_). Reprinted with permission from [70]. Copyright (2022) Springer Nature. (**d**) Mechanical control. Image showing the manual operation of a pushbutton-activated microfluidic dropenser (PAMD) that generates and dispenses droplets. Image showing the droplets generated by the PAMD. Schematic of the composition of the single droplet generation channel and the pumping unit. Reprinted with permission from [71]. Copyright (2021) Elsevier.

Droplets are generated in microfluidics because of fluid instability. This generation employs two immiscible phases, mostly oil and aqueous solutions [40,41]. A droplet is created when two immiscible fluids are simultaneously injected into a microchannel [42,43]. The droplet generation process depends on the fluid viscosity, flow velocities, wetting properties, and geometric features, and it can be classified into passive and active droplet generation [44,45]. Passive droplet generation methods rely on pressure-driven flow and channel geometry, whereas active droplet generation requires external aid from several active control sources [46,47]. Active methods have many advantages over passive methods, including high control of the droplet size and a short droplet generation time [48,49,50]. Active droplet generation can be achieved by controlling additional and intrinsic forces [16]. In general, microfluidic-based droplet generators use various techniques, such as co-flowing [51,52], flow focusing [53,54], crossflow [55,56], electric force [57,58], thermal control [16,59,60], magnetic force [61,62], centrifugal force [63,64], and mechanical control [65,66,67] (Figure 1).

Among these techniques, flow-focusing and crossflow geometries have been widely applied for droplet generation. A flow-focusing microfluidic device is recommended for generating small double-emulsion droplets with a high throughput [72]. Crossflow geometry, whose structure is usually implemented as a T-junction or with another angle (Y-junction) [73,74], is the most common and simplest method for producing droplets. For example, Wu et al. suggested a temperature regulation system for a six-way junction-type flow-focusing microfluidic chip for double emulsion droplet formation using a thermoelectric cooler as the temperature controller [75]. This device could produce double emulsion droplets in the temperature range of 0–40 °C with a high monodispersity. Majd et al. studied the effect of geometry on droplet production and electric field intensity using a flow-focusing 3D-printed microdevice and DC electric field [76]. They found that this system was suitable for generating smaller droplets without significantly altering the frequency of droplet formation. Kieda et al. described a droplet microfluidic platform to generate encapsulated spheroids of MCF-7 breast cancer cells at a flow-focusing junction [77]. The authors introduced an oil-free droplet microfluidic platform for droplet generation, which helps to avoid the potential cytotoxicity of the oil phase. Using 3D drug testing, cancer cells treated with doxorubicin showed dose-dependent cell death after 48 h. Chen et al. developed a homemade pipette droplet microfluidic kit for droplet generation [78]. With the use of 3D printing, the 3D-printed case can be used as a simple tip deformation tool for droplet generation. Lashkaripour et al. suggested a web-based tool, design automation of fluid dynamics (DAFD), that enables the design automation of microfluidic droplet generation with a diverse set of fluids using a flow-focusing technique without requiring extensive machine learning knowledge or large-scale datasets [73]. The authors suggested that this tool delivered a user-specified performance within 4.2% and 11.5% of the desired diameter and rate, respectively. Magnetic control offers noncontact generation of droplets [79,80,81]. Generally, ferrofluids, which are magnetic nanoparticles suspended in an aqueous solution, are employed. Tan et al. introduced a water-based ferrofluid that acted as a dispersed phase for droplet generation [82]. In mechanical control, many methods are employed to physically deform the liquid interface for droplet generation including hydraulic, pneumatic, and piezoelectric actuators [83,84]. For example, Zhang et al. proposed an automated approach using a commercial array spotter for the simple and rapid generation of high-diversity droplet libraries [85]. This device successfully generated droplet volumes ranging from 50 to 800 pL using a piezoelectric droplet generator and required only a few hours to create millions of droplets of hundreds of reagents. The instrument served as an automated droplet generator, allowing the execution of droplet reactions without the need for microfluidic expertise. Xue et al. developed and tested a novel active droplet generator based on a V-valve construct for femtoliter (fL)-volume droplet generation using pneumatic valve generation [86]. This device can produce a single droplet as small as 125 fL and subsequently merge it with an nL-volume sample droplet to generate a sequence of output droplets. Moreover, this platform can be used immediately to maximize the information content associated with single-cell microfluidic experiments, thus representing a flexible and cost-effective tool for high-precision screening applications. Nagesh et al. also used a T-junction microdroplet generator combined with pneumatic actuation to control the droplet size [87]. Table 1 presents a comparison of different droplet generation techniques using the microfluidic approach.

### 2.3. Droplet Manipulation

**Figure 2:**
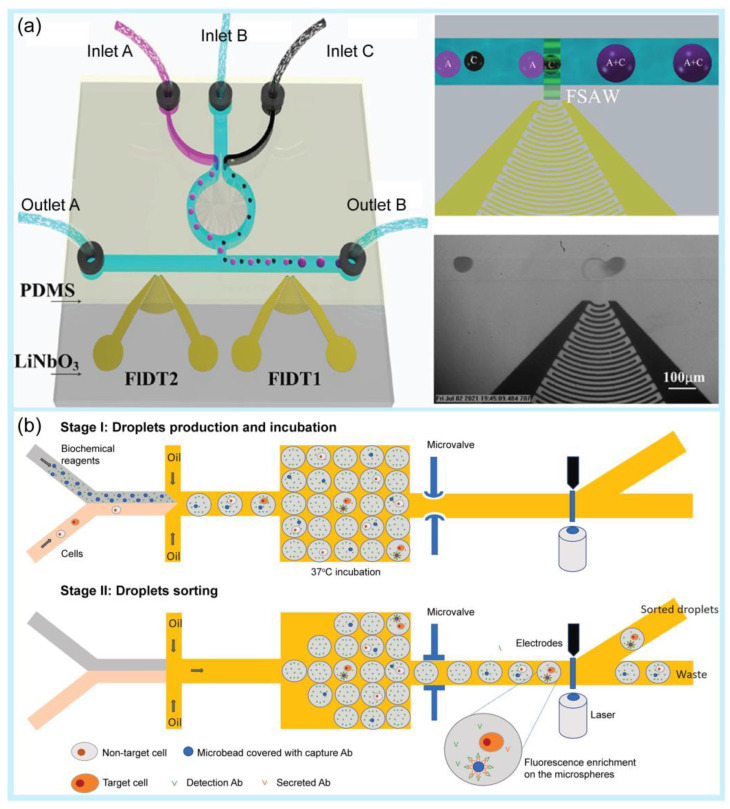
Examples of droplet manipulation. (**a**) Droplet fusion. Schematic of the microfluidic device based on focused surface acoustic waves (FSAW) as an acoustics-controlled fusion method of microdroplets and microbubbles. Reprinted with permission from [108]. Copyright (2022) ACS publications. (**b**) Droplet sorting. Schematic of single-cell sorting using an integrated pneumatic valve droplet microfluidic device. Reprinted with permission from [117]. Copyright (2023) Elsevier.

After droplet generation, the droplets require further manipulation to realize chemical and biological reactions [97,98]. Droplet manipulation includes fission [99,100], fusion [101], mixing [102], sorting [103,104,105], and trapping [106]. Generally, droplet fission reduces the volume of the droplet and controls the chemical concentration encapsulated inside the droplet by producing a high-throughput droplet, whereas droplet fusion allows the introduction of new reagents into the droplet or the merging of droplets with different contents for further application [107]. For example, Jin et al. used an auto-controlled fusion method for microdroplets by utilizing a focused surface acoustic wave [108] (Figure 2a). This approach enabled the fusion of the same or different types of microdroplets with a simpler operation compared with other fusion methods. In another study, Fallah et al. suggested the use of an asymmetric electric field in a T-junction to split and sort droplets simultaneously [109]. Using electrical fields, the size and breakup speed of droplets can be controlled numerically, which is a suitable approach for actively adjusting the size of the droplets. Qin et al. suggested the use of acoustic valves to control the droplets by trapping or releasing them into specific channels [110]. This technique does not use a high voltage or target samples, making it more suitable for microfluidic applications in biochemical experiments.

In addition, mixing the reagents trapped inside the droplet is crucial for a rapid and effective reaction, which can be achieved using dielectrophoretic or piezoelectric actuation [111]. For example, Peng et al. reported an active droplet manipulation strategy based on a slippery magnetically responsive micropillar array for unidirectional droplet transport, multi-droplet transport, droplet mixing, and droplet screening, which is a promising platform for intelligent droplet manipulation [112]. In another study, Chen et al. introduced a magnetism-based method to promote automatic, rapid, and efficient droplet mixing on ‘open-surface’ microfluidics, with the capability of large-scale integration and contamination exemption [113]. Qi et al. introduced a new method based on mechanically activated droplet manipulation using repeated patterns with surface gradient wettability [114]. The droplets were moved on an open surface by arranging repeated patterns with gradient surface wettability and applying symmetric in-plane vibrations, which realized droplet mixing and selective manipulation of multiple droplets. Samlali et al. used a droplet-based microfluidic system for long-term incubation under low-voltage conditions to sort filamentous fungi based on their enzymatic activities [115]. The authors demonstrated that the incubation of single spores in droplets was possible over multiple days (2–4 days), and that this system could be sorted without droplet breakage.

Droplet sorting is the process of distributing droplets into separate downstream channels that can be precisely selected for a further analysis. For example, Zhang et al. presented a convenient method for gathering, splitting, merging, and sorting microdroplets using dynamic pneumatic rails in double-layered microfluidic devices, which was successfully used to sort single-cell droplets [116]. Recently, Zhou et al. introduced an integrated pneumatic valve droplet microfluidic chip for incubation and fluorescence sorting of hybridoma cells [117] (Figure 2b). Using a microvalve, this microdevice could realize the generation, storage, incubation, and sorting of droplets, and the purity of the droplets was approximately 89.7% after sorting using a fluorescence-activated droplet sorting platform. Therefore, it significantly minimizes cell loss and reduces the risk of droplet fusion.

### 2.4. Open Droplet Microsystem

**Figure 3:**
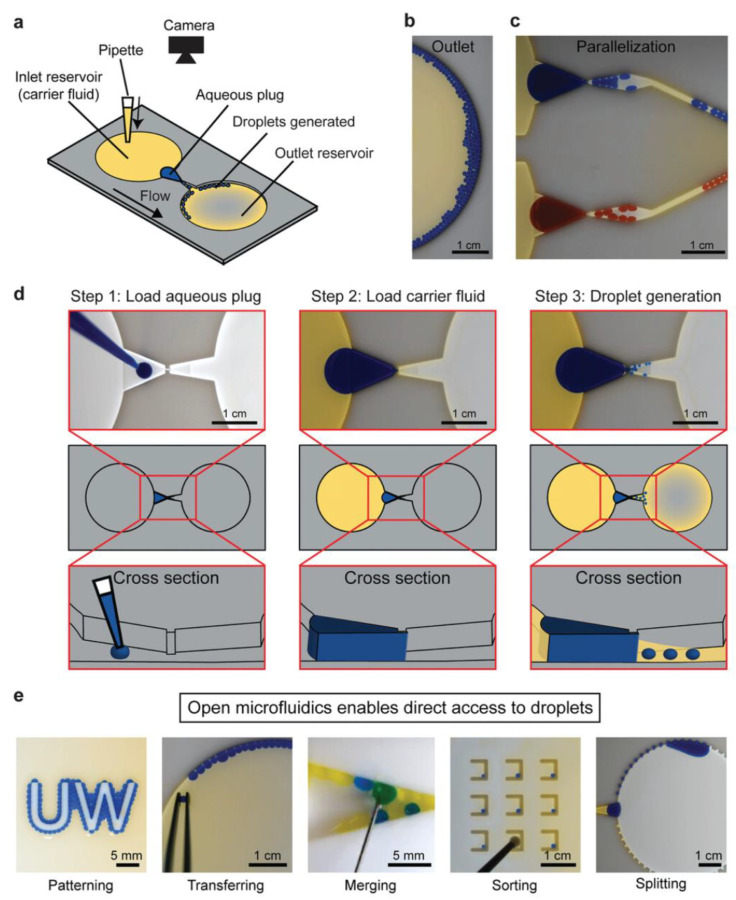
Example of open droplet microfluidic. (**a**) Schematic of the device, (**b**) image of generated droplets in the outlet reservoir, and (**c**) droplets generated in parallel. (**d**) Workflow for droplet generation using passive forces derived from pressure. (**e**) Downstream droplet manipulations. Reprinted with permission from [120]. Copyright (2021) Wiley.

Typical microfluidic structures are closed chambers and channels, and the fluid inside them is driven by valves, pumps, junctions, and tubing. Open microfluidic devices have at least one face open to the environment [118]. Open droplet microfluidic systems combine the advantages of conventional microfluidic systems with an ease of use [118]. Direct droplet generation and on-demand droplet manipulation allow easy handling by users without the need for complicated instruments such as pump systems. Open droplet microfluidic systems can be classified as static and dynamic systems. In static systems, hydrophilic patterns are typically modified on the device surface. Droplets are generally generated and located in these hydrophilic regions. The hydrophobic regions serve as the boundaries. Dynamic systems offer more flexible droplet manipulation methods than static systems. Droplet manipulation can be controlled by modifying the device geometry and surface [118]. Research has introduced and developed new designs for open droplet microfluidics and employed them in many applications. A spheroid culture plate in the form of 384 hanging drop arrays was used to observe drug action modes in physiological 3D cell spheroids [119]. Recently, Khor et al. demonstrated an open-channel droplet microfluidic system for generating droplets down to 100 nL at low capillary numbers with a power-free, enhanced usability, a compact footprint, and a high customizability (Figure 3) [120]. A fast multiscale liquid-patterning method was introduced on open surfaces [121]. A simple pillar array was used to control the droplet shape and size. The shear force was enhanced by the microstructures. When an elastic sweeper was applied, small droplets were generated and trapped in the microstructure. Open droplet microfluidic platforms offer many advantages for biological and chemical applications, including simple fabrication and operation. Compared with closed systems, the designs of these microfluidic devices are flexible and unique in 3D geometries. Moreover, they can handle liquids without the need for liquid-handling equipment and pipettes using a simple and low-cost approach. In addition, open systems make it easy to access the droplet content at any time, whereas in closed systems, droplets can be achieved at the opening locations of the systems. This characteristic can benefit a cell culture, making it easy to monitor changes in cells in real-time. However, there are still some challenges, such as a low throughput and difficulty in controlling evaporation. Overall, an open droplet microfluidic system offers many capabilities when employed in appropriate applications.

## 3. Droplet-Based Microfluidic Technology for Pharmaceutical Applications

### 3.1. Drug Synthesis

**Figure 4:**
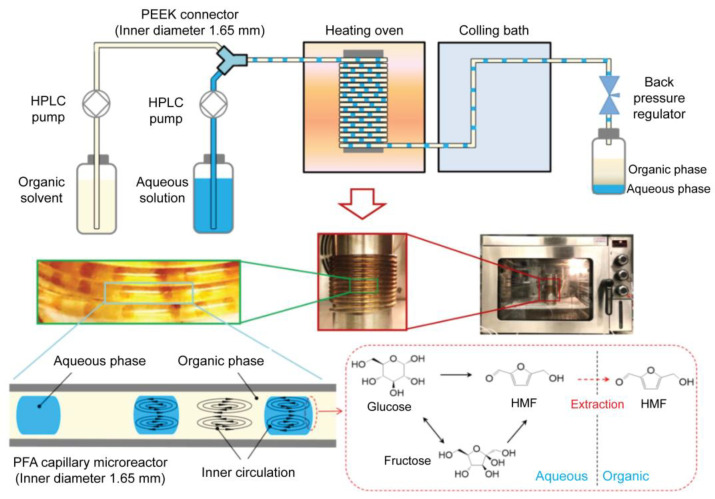
Schematic and optical images of the biphasic slug flow microreactor for in situ synthesis of HMF. The inner circulation in droplets and slugs promoted mixing/reaction in the aqueous phase and enhanced in situ extraction of HMF to the organic phase. Reprinted with permission from [127]. Copyright (2020) Elsevier.

Drug synthesis is the first and most important step in drug development. For drug synthesis, it is essential to identify lead compounds. Lead compounds can be separated and purified from natural sources, chemically synthesized, or molecularly modeled. The synthesis proceeds via chemical processes for lead compound optimization after lead compound selection. Generally, these chemical reactions occur in bulky systems; therefore, they still have limitations, such as a large reagent consumption, long reaction times, and difficulty in synthesis monitoring. Microfluidic technology, particularly droplet-based microfluidic technology, has been extensively employed to overcome these shortcomings [122]. The application of droplet-based microfluidic devices in drug synthesis has many advantages, including reduced reagent and reaction times, a high throughput, reproducibility, and improved control [19,123]. In this section, we review studies on how droplet-based microfluidics can be employed in drug synthesis. A previous study introduced a method for producing solid particles via indigo synthesis [124]. In some chemical synthesis processes, the production of lead compounds can cause channel clogging. Solid products from chemical synthesis were successfully collected from the walls along the tubing using monodisperse droplets in a single reactor, and the synthesized products were confined inside these droplets. This strategy helps to control the chemical synthesis process and increase synthesis efficiency. A novel inert 3D emulsification device was designed for pharmaceutical applications [125]. Nanosized drug particles can help bring out specific surfaces of the particles, leading to easy absorption inside the body. In this research, the authors introduced a chemically inert and stable device, which is an ideal platform for nanosuspension preparation of poorly water-soluble active pharmaceutical ingredients. A 3D flow-focusing device was fabricated using glass. Glass offers an inert emulsification platform for solvents and drug particles, where no chemical reactions occur with the wall or substance absorption through the wall. In this study, fenofibrate was used as a model pharmaceutical ingredient because of its low water solubility. Each droplet contained exactly one particle. Fast supersaturation occurred, leading to the formation of monodisperse nanoparticles. The particles had diameters as small as 128 nm, and the particle concentration increased by 250 times. A droplet-based microfluidic system was introduced to perform an ionic liquid (IL)-based Suzuki–Miyaura cross-coupling reaction [126]. To generate uniform droplets, a FC-40 fluorocarbon-based liquid was employed as the continuous phase to disperse the reactants. The FC-40 fluorocarbon-based liquid offers many advantages, including preventing microchannel clogging and increasing the synthesis efficiency within 10 min of the reaction process. Small itraconazole nanoparticles were produced using a droplet-based microfluidic device. This system offers an ideal mixing environment for controlling the size and uniformity of the droplets. Smaller nanoparticles are obtained using this method. Recently, Guo et al. proposed the synthesis of 5-hydroxymethylfurfural (HMF) from glucose in a biphasic system in a slug-flow microreactor using a combination of AlCl_3_ and HCl as catalysts (Figure 4) [127]. They found that HMF yields of over 66% were obtained from 1 M of glucose in 16 min under optimized conditions; thus, with the use of a microreactor, the HMF yields were higher than those in a batch reactor, which shows a high potential application for more efficient and sustainable HMF synthesis from glucose.

### 3.2. Drug Screening

**Figure 5:**
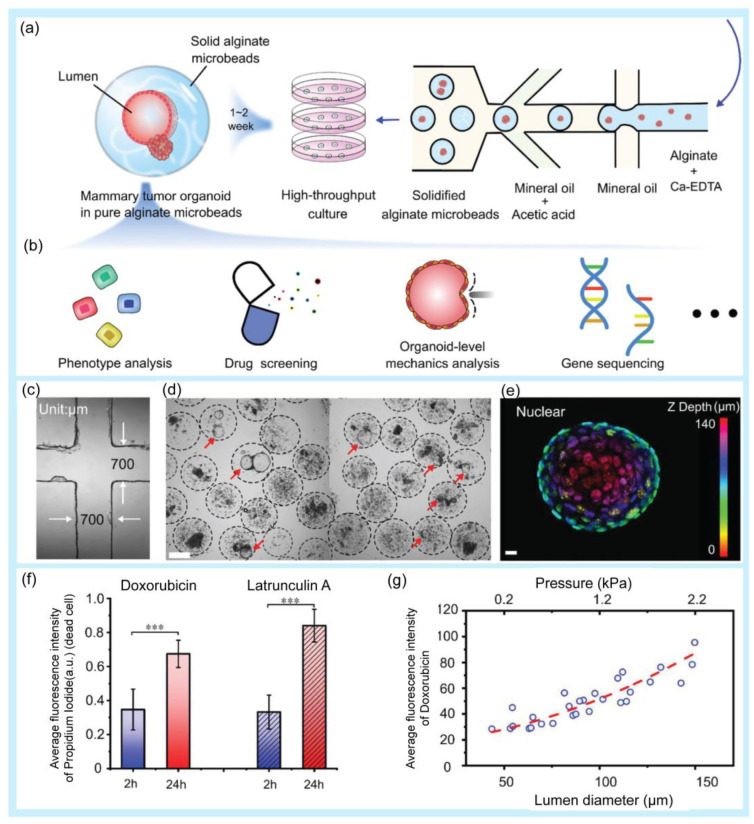
Examples of droplet-based microfluidic applications in drug screening. (**a**) Schematic of high-throughput generation of alginate microbeads with tumor pieces encapsulated by the microfluidic droplet technique. (**b**) Potential application of the mammary tumor organoids in alginate microbeads. (**c**) Microfluidic chip channel under a 4× objective lens. (**d**) High-throughput generation of mammary tumor organoids in alginate microbeads. Scale bar: 200 µm. (**e**) 3D schematic of a 140 µm luminal organoid: nuclear mapping within the *Z*-axis (upper image) and cross-sectional view at *z* = 63 µm (**bottom**, **right**). Bright-field image of the organoid (**bottom**, **left**). Scale bar: 10 µm. (**f**) Fluorescence intensity of dead cells after the drug treatment. The values were normalized against the highest intensity (mean ± SD, *n* = 10, *** *p* < 0.001). (**g**) Correlation of doxorubicin uptake, organoid size, and luminal pressure. Reprinted with permission from [136]. Copyright (2021) Wiley.

Drug screening is the process of identifying promising candidates and determining the correct concentration from a large amount of natural and synthetic drug compounds [6]. In the first round of drug screening, lead compounds are identified. The screening process continues with additional pharmacological and physicochemical reactions to select lead compounds. The lead compounds then undergo additional screening steps before undergoing clinical trials [128]. Droplet-based microfluidics have provided an effective approach for drug screening [17,129,130,131]. A high-throughput platform for screening natural antibiotics has been developed [132]. Hydrogel microdroplets are used in this approach. Both bacteria-*Staphylococcus aureus* (*S. aureus*) and recombinant antibiotic-producing microbes, including *Saccharomyces cerevisiae* and *E. coli*, are co-emulsified. Antibiotic susceptibility tests were performed using fluorescent dyes. The screening process successfully selected antibiotics secreted by a wide range of yeasts. Moreover, the introduced platform was used to screen a metagenomic library of antibiotics against *S. aureus*. A droplet microfluidic platform that can mix, trap, and release droplets was developed to screen drug compounds with potential activity against the AcPHF6 tau-hexapeptide [133]. The tau-peptide aggregation is known for its relation to Alzheimer’s disease. The screening assay using a droplet microfluidic platform has many advantages in comparison with conventional 96-well plate studies, including reagent saving and a fast reaction time, from 2 h for the conventional method to 2.5 min. Research based on droplet microfluidics has been conducted to rapidly screen *Streptomyces* [134]. *Streptomyces* is a Gram-positive filamentous bacterium. This genus of bacteria is usually found in the soil. Actinobacteria, especially *Streptomyces*, produce more than 60% of existing antibiotics. The use of a droplet-based microfluidic platform improves some of the limitations of conventional methods for bacterial screening, including a low efficiency and long analysis times. This approach can successfully screen *Saccharomyces* in mycelial form and extracellular products such as pharmaceuticals, whereas conventional screening methods cannot. A large-scale droplet array was introduced for high-throughput screening applications [135]. The large-scale two-dimension droplet array was formed using a microcage array chip. Using this platform, up to 1,000,000 droplets were generated within a few seconds. These findings have the potential for drug screening. Fang et al. used microfluidic droplet technology for high-throughput generation of tumor organoids in alginate microbeads (Figure 5) [136]. In this study, two types of organoids (luminal and solid) were formed from mouse tumor pieces and cultured inside non-adhesive alginate microbeads for over 21 days with high cell viability. For application in a drug screening platform, tumor organoids were treated with doxorubicin and latrunculin A, and the results showed that the drug response was related to the luminal size and pressure of the organoids.

**Figure 6:**
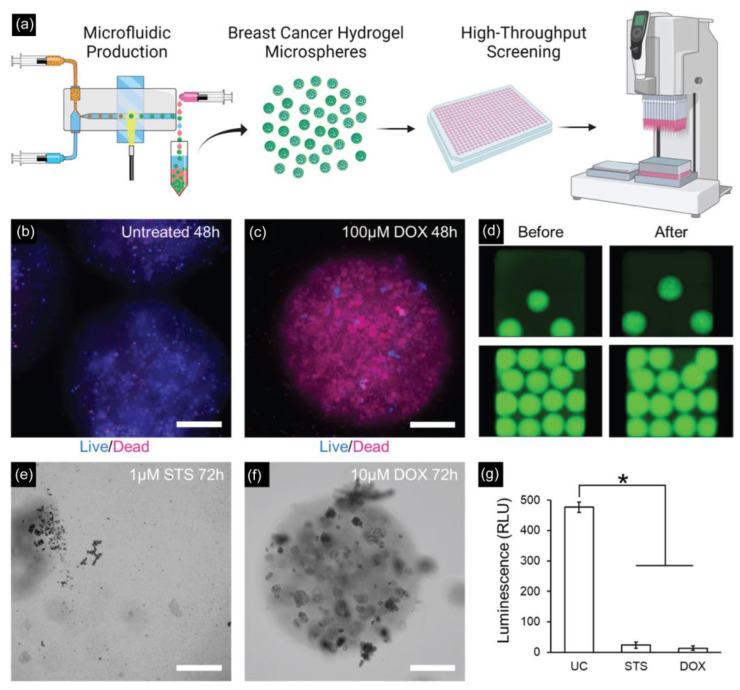
(**a**) Schematic of high-throughput generation of hydrogel microspheres with tumor pieces encapsulated by the microfluidic droplet technique for 3D culture and tumorigenic characterizations. (**b**,**c**) Drug testing results on MDA-MB-231 microspheres with untreated and treated microspheres with 100 μM doxorubicin for 48 h. Scale bar: 200 µm. (**d**) Fluorescent images of MDA-MB-231 microspheres before and after spinning for delivering drugs to the assay plate. (**e**) MDA-MB-231 microspheres treated with 1 μM staurosporine for 72 h completely degraded. Scale bar = 200 μm. (**f**) MDA-MB-231 microspheres treated with 10 μM doxorubicin are mostly intact but with visible cell damage. Scale bar = 200 μm. (**g**) Assessing the viability of MDA-MB-231 microspheres after 72 h of incubation (* *p* < 0.05, *n* = 5 wells per group). Reprinted with permission from [137]. Copyright (2022) ACS publications.

Recently, Seeto et al. fabricated hydrogel microspheres (submillimeter range) using a facile droplet microfluidic approach for potential applications in high-throughput screening assays (Figure 6) [137]. By using a microfluidic PDMS device with a modified T-junction design, a PEG-fibrinogen-based hydrogel microsphere (800–1000 μm in diameter) containing two breast cancer cell lines (MCF7 and MDA-MB-231) was obtained for a 3D culture and characterized in terms of viability, metabolic activity, and proliferation over 14 days in culture.

A droplet-based microfluidic platform has been developed to evaluate the effectiveness of drug administration in monocytic THP-1 cells [138]. Each droplet served as a bioreactor to track the cell viability under three types of drug treatments: temsirolimus, BAY 11-7082, and rifabutin. Cell experiments carried out inside the droplet minimized contamination. The link between inflammasomes and apoptosis in tumor cells has been fully explained, which offers the potential for further studies on drug development. With the aid of microfluidic technology, the need for equipment and machinery has been eliminated, and the cell evaluation process has been simplified. This study provides strong evidence that ASC, a bipartite protein, plays an important role in communication between apoptosis and inflammasomes.

### 3.3. Drug Delivery

**Figure 7:**
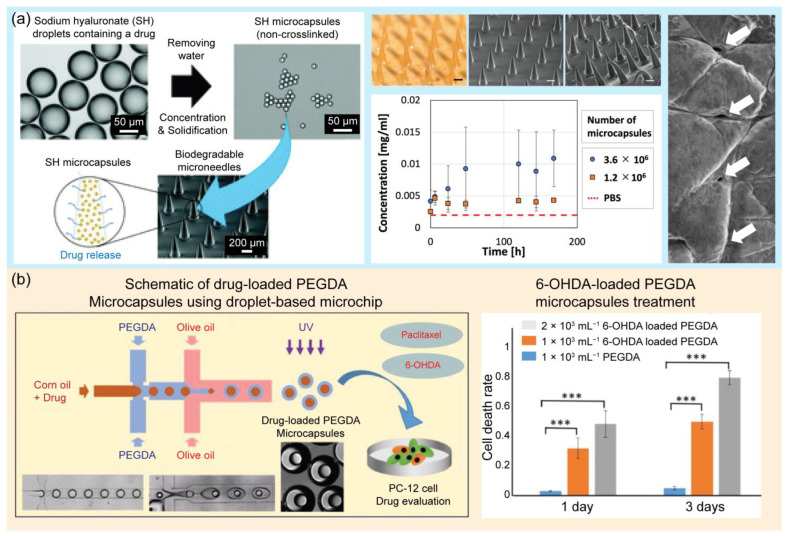
Examples of droplet-based microfluidic applications in drug delivery. (**a**) Schematic of biodegradable microneedles (MNs) embedded with drug-containing microcapsules that release the drug over time. The photograph shows the MNs containing microcapsulates, and the graph shows the concentration of FITC-BSA in PBS over time, that is, the time course of cumulative release to PBS. Surface SEM image of the porcine skin surface after insertion and removal of the MNs. Reprinted with permission from [151]. Copyright (2021) Royal Society of Chemistry. (**b**) Schematic of the preparation of drug-loaded PEGDA microcapsules using a droplet-based microchip and its application in drug evaluation of PC12 cells. The graphs show the results of PC12 cells after paclitaxel-loaded PEGDA and 6-OHDA-loaded PEFDA microcapsule treatment for 1 and 3 days. *** *p* < 0.001; *n* = 9. Reprinted with permission from [160]. Copyright (2022) Elsevier.

In another application, droplet-based microfluidics have been widely adopted for the synthesis of drug-loaded microcapsules for drug delivery, including site targeting and controlled release, in tumor therapy and pharmacological research [139,140,141]. Delivery is an important aspect of drug development. Drug delivery aims to administer a drug to a specific site in the body to treat a disease or relieve symptoms. Oral, parenteral, inhalation, gastrointestinal, and transdermal administration are the common approaches for drug delivery [139]. These routes of administration are effective but still have many limitations, including difficult control of quantity and quality, an instability of drugs in in vivo environments, and side effects [142,143]. Alternatively, droplet-based microfluidic platforms for drug delivery have been extensively developed [144,145]. These approaches can help control the delivery and release of appropriate concentrations of drugs to designated sites in the body [146,147]. In this section, several applications of droplet-based microfluidic platforms for drug delivery are reviewed. Droplet generation devices based on glass capillaries and flow-focusing droplet generation methods have been introduced to control drug release [148]. The size and morphology of microparticles are important factors for drug delivery. Droplet-based microfluidics offer an excellent tool for controlling monodisperse microparticles and the desired morphology [149], and this study suggested a non-PDMS tube-in-tube glass capillary microdevice for the generation of drug-loaded microcapsules. Glass capillary devices have many advantages, including a simplicity of fabrication, reusability, a high throughput, a low cost, and easy control of the droplet morphology and size. This study proved that drug release kinetics are strongly affected by the size and morphology of poly(lactic-co-glycolic acid) (PLGA)-loaded particles. PLGA is a promising biodegradable material for biomedical applications. It is a Food and Drug Administration (FDA)-approved polymer that has been extensively employed to develop drug delivery systems for many substances such as small-molecule drugs, chemotherapeutics, and proteins [150]. These results provide useful guidelines for the production of microcapsules. A previous study developed a new approach for preparing biocompatible and water-soluble sodium hyaluronate monodisperse microcapsules for transdermal drug delivery systems [151]. Instead of cross-linking methods, shrinkage and gelation techniques were applied in this study to remove water from monodispersed drug-containing SH droplets. Microcapsules are water-soluble; therefore, they stably dissolve and release the drug into the skin. Moreover, they can be stored for up to 1 month. Monodisperse microcapsules were embedded in PLGA-based microneedles for transdermal drug delivery. The application of this novel material can prevent the issues caused by common cross-linking methods, including bursts. In the presence of water, cross-linking gels create fragile structures that easily burst and release drugs discontinuously. Chen et al. introduced an approach using magnetic thermosensitive hydrogels for controlled drug release [94]. Magnetic thermosensitive hydrogels with core–shell structures were prepared using a droplet-based microfluidic platform. Two types of anticancer drugs, hydrophobic and hydrophilic, were loaded into the core and shell of the double emulsions to achieve simultaneous delivery. Fe_3_O_4_ nanoparticles were embedded inside the shell layer of the emulsion to achieve magnetic-based delivery to specific targets. Moreover, the thermal effect of the nanoparticles caused an increase in the hydrogel temperature. Consequently, the swelling state of the hydrogel changed, leading to the release of the drug to kill the selected tumor cells. Magnetic thermosensitive hydrogels have many advantages in the field of tumor treatment because they can achieve a dual response and magnetic hyperthermia. This approach offers on-demand and controlled drug delivery by controlling the magnetic field strength, exposure time, and amount of loaded nanoparticles. One study employed a droplet-based microfluidic device to synthesize polymeric nanocarriers [152]. Nanogels were generated using a flow-focusing microfluidic device. Nanogels are potential candidates for drug delivery because they can encapsulate both lipophilic and hydrophilic substances, reduce drug doses, and decrease side effects. Hydrogels are networks of three-dimensional cross-linked polymers that can absorb large amounts of water [153,154,155]. Physical and chemical methods are typically employed to synthesize hydrogels via polymer cross-linking. Hydrogels with good mechanical strength and biocompatibility can be used in pharmacology. Polymeric hydrogels modified with active chemicals offer bioinert matrices and improve the regulatory and transport functions for drug delivery [156]. In this study, linear polyethyleneimine and hyaluronic acid were used to synthesize nanogels. Furthermore, using this system, doxorubicin, a chemotherapeutic agent, was successfully delivered to target tumor cells. A microfluidic chip was developed to produce chitosan–oil–chitosan double emulsions for drug delivery [157]. Chitosan, a polycationic polysaccharide with excellent bioactivity and biocompatibility, has been widely used in biomedical fields, including tissue engineering and drug delivery. In this study, microfluidic chips were developed to assist in the generation of chitosan microparticles of a uniform size and monodispersity. An intelligent hydrogel-based child–parent microrobot was developed to deliver drugs to the small intestinal sites [158]. The small intestine is a potential drug loading site. However, some challenges still exist; the drug must be resistant to the harsh conditions in the stomach before entering the small intestine, and the drug concentration must be maintained for the expected time. Microrobots have gained immense attention for drug delivery applications. There are four shapes of microrobots, including teardrops, spheres, mushrooms, and red blood cells, which can affect the release efficiency. Chemical and magnetic sources are the two main energy sources that microrobots convert into mechanical motion. In this study, hydrogel-based child–parent microrobots of four shapes were created using an extrusion-dripping method. Vision-feedback magnetic driving systems are employed to guide the microrobots to their destinations. Child–parent microrobots loaded with doxorubicin hydrochloride exhibit an anticancer cell (Hepa 1–6) ability. The proposed system offers a promising solution for the oral administration of small intestine-targeted therapy. A study was conducted to synthesize supramolecular microgels using a droplet-based microfluidic device [145]. The synthesized microgels exhibited pH-responsive properties. The encapsulation and pH-triggered release ability of the introduced microgels offer high potential for molecular delivery and release at the desired target. In this study, supramolecular microgels were synthesized using a natural crosslinker, tannic acid, for an amphiphilic stimuli-responsive polymer, poly(N-vinylcaprolactam), in droplets. Droplet-based microfluidics were employed to generate monodisperse droplets, which served as templates for pH-responsive and degradable supramolecular microgel synthesis. The response of the microgels to different pH conditions was also evaluated. At a high pH, the microgel rapidly degraded because of the disruption of hydrogen bonds. Under acidic conditions, the microgel deswelled, owing to the protonation of phenolic groups in tannic acid and an increase in hydrogen bonds. This microgel characteristic is useful for encapsulation and release applications, particularly in delivery systems. The main challenges in this study were to avoid blocking the microchannel during the gelation process and ensure the stability of the microgel network. Chen et al. proposed, in 2023, a microfluidic approach for the local treatment of gastric cancer [159]. Bubble microcapsules were generated using a microfluidic system to load drugs. PLGA was used as the shell for the double emulsion droplets because of its selective water permeability. When the microcapsules were treated with the hypertonic salt solution, the water inside the core was drained. This results in the formation of bubbles in the microcapsules. Bubble microcapsules are excellent drug carriers that overcome barriers in the gastrointestinal tract and prolong the effective time of the drug. In addition, PLGA has high encapsulation rates for many types of drugs and can improve drug release. This approach has demonstrated promising applications in cancer treatment. Figure 7 illustrates the applications of droplet-based microfluidic devices for drug delivery.

## 4. Conclusions and Future Perspectives

Droplet-based microfluidics offer new possibilities for drug development; however, some challenges still need to be addressed before widespread application. The first challenge for broader applications is the standardization of device fabrication, instrumentation, and droplet handling. The standardization of these factors can help generate droplets of a uniform size and shape and manipulate droplets with less variation [42]. Fluorescent markers are typically employed in droplets for many applications. In many cases, fluorescence leakage issues occur, resulting in the failure of analytical results in droplet assays. Fluorescent marker selection is important to prevent leakage; for example, resofurin leaks easily through droplets; therefore, it is not suitable for use with long incubation times [161]. Moreover, the stability of droplets in surfactants should be improved to prevent the leakage of reagents and samples. The surfactants used to stabilize the droplets are amphiphilic molecules. Different groups have different affinities for different immiscible phases [24]. To overcome the limitations of common surfactants, new molecules with structures that match the oil and material of the device should be developed. Many microfluidic platforms have been tailored to study the behavior of surfactants and emulsions to improve surfactants. Another limitation is the lack of user friendliness. Thus, there is a need for trained personnel working in droplet-based microfluidic systems. Controlling reagent concentration during assay use is an important and challenging issue. In addition, the question of how to fully recover droplets and droplet storage and reservations still demands considerable effort from scientists. In 2022, the FDA authorized 37 new drugs in the United States. Despite huge efforts in new drug discovery, the number of new FDA-approved drugs is still very limited. Recently, with the approval of 3D-printed drug products, the FDA has encouraged the application of advanced technologies, including 3D printing and microfluidics, in drug discovery [162]. Droplet-based microfluidics have shown great potential for pharmaceutical applications. The application of this technique can be further enhanced by improving the device design, material, and chemical and biological functions. The FDA has built a library of approved drugs that can be reversibly immobilized on beads. This can be a great guide for the employment of a one-bead-one-compound in microfluidics for drug discovery [163,164].

## Figures and Tables

**Table 1:** Comparison of different droplet generation techniques using microfluidic devices.

Type of Control	Droplet Generator	Fabrication Technique	Advantages	Disadvantages	Ref.
Thermal control	Flow focusing	Soft lithography (*)	High monodispersity Controllable size	Requires a temperature control system	Wu et al., 2023 [75]
Electrical control	Flow focusing	Soft lithography (*)	Fast response Suitable for cell studies	Requires high voltages	Jiang et al., 2022 [88]
High-pressure tetrafluoride pump control	Step-emulsification	CNC milled microdevice (*)	Fast, high throughput Self-assembly of droplets into 3D and 2D arrays	Design dependence Requires optimization	He et al., 2022 [89]
Centrifugal control	Dispenser nozzles	Glass capillary nozzles (**)	High throughput Mass production Suitable for cell studies and drug delivery	Requires a rotator system. Complex and costly Design dependence	Li et al., 2022 [90]
Pressure syringe pump control	Crossflow (T-junction)	Soft lithography (*)	Simple actuation Suitable for biology samples	Requires a specific flow rate Bubble generation, evaporation	Li et al., 2023 [91]
	Flow focusing	Soft lithography (*)	Simple actuation Suitable for cell studies	Requires a specific flow rate	Fevre et al., 2023; Luo et al., 2023 [92,93]
	Crossflow (T-junction)	CNC milled microdevice (*)	Suitable for drug delivery (anticancer drugs)	Low monodispersity	Chen et al., 2022 [94]
	Co-flowing	3D printing and cylindrical glass capillary (**)	Suitable for surfactant adsorption	Requires pressure sensors Design dependence	Liang et al., 2022 [95]
Gravity force	Co-flowing	3D printing (**)	No use of power Controllable size	Requires a vertically placed microdevice Design dependence	Wu et al., 2023 [96]

3D: three-dimensional; 2D: two-dimensional; (*) 2D device; (**) 3D device; CNC: computer numerical control.

## Data Availability

No new data were created or analyzed in this study. Data sharing is not applicable to this article.
